# The synergistic effect of minocycline and azole antifungal drugs against *Scedosporium* and *Lomentospora* species

**DOI:** 10.1186/s12866-021-02433-6

**Published:** 2022-01-12

**Authors:** Fang Yang, Yi Sun, Qiaoyun Lu

**Affiliations:** 1 Department of Dermatology, Shenzhen People’s Hospital (The Second Clinical Medical College, Jinan University, The First Affiliated Hospital, Southern University of Science and Technology), Shenzhen, 518020 Guangdong China; 2 Candidate Branch of National Clinical Research Center for Skin Diseases, Shenzhen, China; 3 Department of Dermatology, Jingzhou Central Hospital, The Second Clinical Medical College, Yangtze University, Jingzhou, Hubei China; 4 Xiangyang Central Hospital, Affiliated Hospital of Hubei University of Arts and Science, Xiangyang, Hubei China

**Keywords:** *Scedosporium*, *Lomentospora*, Minocycline, Azole, Synergy, Antifungal effect, Resistance

## Abstract

**Background:**

This study was aimed to determine the potency of Minocycline (MIN) and azoles, including itraconazole (ITR), voriconazole (VOR) and posaconazole (POS) against *Scedosporium* and *Lomentospora* species.

**Results:**

This study revealed that MIN exhibited no significant antifungal activity against any of the tested strains, whereas in vitro combination of MIN with ITR, VOR or POS showed satisfactory synergistic effects against 8 (80%), 1 (10%), and 9 (90%) strains, respectively. Moreover, combined use of MIN with azoles decreased the minimum inhibitory concentration (MIC) range from 5.33–16 μg/ml to 1–16 μg/ml for ITR, from 0.42–16 μg/ml to 0.21–16 μg/ml for VOR, and from 1.33–16 μg/ml to 0.33–16 μg/ml for POS. Meanwhile, no antagonistic interactions were observed between the above combinations. The *G. mellonella* infection model demonstrated the in vivo synergistic antifungal effect of MIN and azoles.

**Conclusions:**

The present study demonstrated that combinations between MIN and azoles lead to synergistic antimicrobial effects on *Scedosporium* and *Lomentospora* species, while showing a potential for overcoming and preventing azole resistance.

## Introduction

The incidence of fungal infections is increasing year by year. In particular, invasive fungal infections are increasingly becoming an important factor endangering human health and life [1]. Invasive fungal infections are predisposed to occur in immunocompromised patients as well as hospitalized patients with severe underlying diseases [2, 3]. *Scedosporium* and *Lomentospora* species are an aggressive and conditional pathogen, causing a rare type of fungal infection, with only 370 cases reported worldwide up to 2007 [4]. The infection involves a variety of sites and is relatively difficult to be diagnosed clinically because it shares a great similarity in clinical characteristics and histopathology with aspergillosis, infection with *Fusarium*, and other relatively common hyalohyphomycosis [5].

Azoles, including itraconazole (ITR), voriconazole (VOR) and posaconazole (POS), are an earlier class of drugs used to treat fungal infections [6]. According to epidemiological investigation, the prevalence of *Scedosporium* and *Lomentospora* species is increasing, posing a serious threat to the effectiveness of antifungal drugs [7]. Liu et al. showed that *Scedosporium* and *Lomentospora* species are highly resistant to antifungal drugs, highlighting the need for alternative treatment modalities [8]. A recent study showed that Lomentospora prolificans and Scedosporium apiospermum were well tolerated by POS in combination with terbinafine [9]. Moreover, combination antifungal therapy with VOR has been demonstrated to be a promising treatment option for invasive, *Lomentospora prolificans* infections [10]. Collectively, these observations indicate that combination therapy could be an important clinical treatment strategy for this kind of refractory infection.

Minocycline (MIN) is a semisynthetic broad-spectrum tetracycline antibiotic that displays a similar antibacterial spectrum with tetracycline. MIN can combine with tRNA to achieve antibacterial effect, while being the most potent antibacterial agent among the existing tetracycline antibiotics. Moreover, previous studies have demonstrated that Azoles (fluconazole) in combination with MIN is a potential approach for counteracting *Candida albicans*- *Staphylococcus aureus* dual-species biofilms [11]. Based on these facts, we propose that MIN may have a certain antifungal effect, and as an additive, it may enhance the efficacy of commonly used azoles against *Scedosporium* and *Lomentospora* species.

## Results

### In vitro interactions between MIN and azoles against *Scedosporium* and *Lomentospora* species

As presented in Table 1, MICs for MIN were ≥ 16 μg/mL for all strains, while MICs for ITR were ≥ 16 μg/mL for 8 out of the 16 strains and ranged from 2 to 8 μg/mL for the rest strains. On the contrary, both VOR and POS displayed a lower MIC, ranging between 0.25 and 4 μg/mL for 10 out of the 12 strains. In the meantime, all drugs had a MIC of ≥16 μg/mL for the two *Lomentospora* strains.

**Table 1:** MIC and FICI results with the combinations of MIN and azoles against *Scedosporium apiospermum*

Strain	MIC (ug/mL) for:						
	Agent alone				Combination [A/B(ug/mL)] (FICI)		
	MIN	ITR	VOR	POS	MIN/ITR	MIN/VOR	MIN/POS
*Scedosporium aurantiacum* *(CBS 116910)*	≥16	8	0.5	2	4/2(S)	4/0.25(I)	8/0.5(S)
*Scedosporium minutispora* *(CBS 116911)*	≥16	2	0.25	2	4/0.25(S)	8/0.25(I)	8/0.5(S)
*Scedosporium boydii* *(CBS 101.22)*	≥16	2	0.25	2	8/1(I)	16/0.25(I)	4/0.5(S)
*Lomentospora prolificans* *(CBS 467.74)*	≥16	≥16	16	≥16	4/8(S)	4/8(I)	4/8(S)
*Scedosporium dehoogii* *(CBS 117406)*	≥16	2	0.5	2	4/1(I)	4/0.5(I)	8/0.5(S)
*Scedosporium apiospermum* *(CBS 116899)*	≥16	4	0.25	2	4/1(S)	4/0.25(I)	8/0.5(S)
*Scedosporium boydii* *(1)*	>16	>16	1	2	8/1 (S)	8/0.25(I)	8/0.5(S)
*Scedosporium boydii* *(2)*	>16	4	1	1	4/1(S)	8/0.25(I)	8/0.25(S)
*Scedosporium boydii* *(3)*	>16	>16	1	4	8/2(S)	8/0.5(I)	8/1(S)
*Scedosporium apiospermum* *(4)*	>16	>16	1	4	8/4(S)	8/0.25(I)	4/1(S)
*Scedosporium apiospermum* *(5)*	>16	>16	1	2	8/4(S)	8/0.5(I)	8/0.5(S)
*Scedosporium apiospermum* *(6)*	>16	>16	0.5	2	8/4 (S)	4/0.5 (I)	8/0.5(S)
*Lomentospora prolificans* *(7)*	>16	>16	>16	>16	16/16(I)	16/16(I)	16/16(I)
*Scedosporium minutisporum* *(8)*	>16	8	1	2	8/2(S)	8/0.25(S)	8/0.5(S)
*Scedosporium apiospermum* *(9)*	>16	>16	0.5	4	8/16(I)	8/0.125(I)	8/1(S)
*Scedosporium boydii* *(10)*	>16	4	0.25	1	4/1(S)	8/0.125 (I)	8/0.25(S)

MIC is determined by the concentrations of drugs at which growth was completely inhibited. FICI results are shown in parentheses. S, synergistic effect (FICI of 0.5); I, no interaction (indifference) (0.5 FICI 4)

Combination of MIN with ITR, VOR, or POS exhibited synergistic activities against 12 (75.00%), 1 (6.25%), or 15 (93.75%) strains of *Scedosporium* and *Lomentospora* species (Table 1). Notably, the combination of MIN and VOR only displayed a synergistic activity against strain *Scedosporium minutisporum* (8)*.* Meanwhile, no antagonistic interactions were detected in each of the tested combinations.

### In vivo interactions between MIN and azoles against *Scedosporium* and *Lomentospora*

**Fig. 1:**
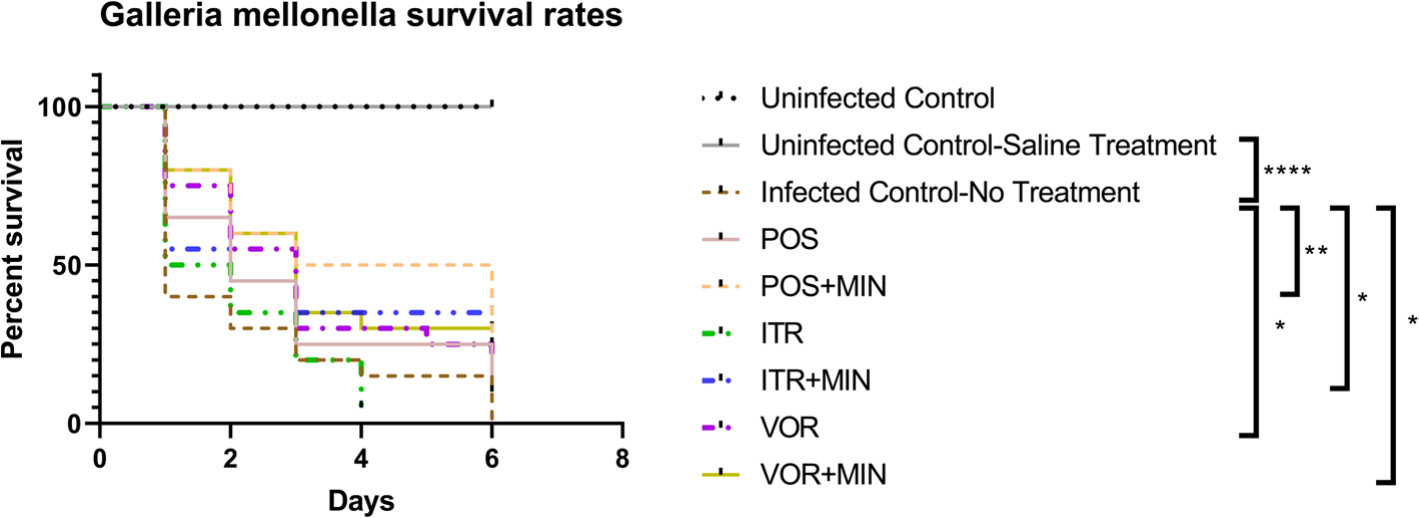
*G. mellonella* survival rates

To assess the synergistic effects of MIN and azoles in vivo, *G. mellonella* were infected with *Scedosporium apiospermum*, and the infected larvae were then treated with MIN and azoles. As shown in Fig. 1, treatment with MIN in combination with POS, ITR or VOR led to a survival rate of 25, 35% or 30%. Moreover, we observed that compared with Infected Control-No Treatment group, VOR treatment slightly increased larval survival, while POS or ITR alone did not significantly increase the survival. By contrast, administration with MIN combined with one of Azoles significantly prolonged larval survival time (*P* < 0.05). Together, these findings demonstrated the in vivo synergistic antifungal activity of MIN and azoles against *G. mellonella*, as evidenced by the increased larval survival.

Uninfected Control Group, wild type larvae without *Scedosporium apiospermum* infection; Uninfected Control-Saline Treatment, wild type larvae injected with saline; Infected Control-No Treatment Group, larvae infected with *Scedosporium apiospermum* receiving no treatment; POS Group, *Scedosporium* infected larvae treated with POS only; POS + MIN Group, *Scedosporium* infected larvae treated with POS in combination with MIN; ITR Group, *Scedosporium* infected larvae treated with ITR only; ITR + MIN Group, *Scedosporium* infected larvae treated with ITR in combination with MIN; VOR Group, *Scedosporium* infected larvae treated with VOR only; VOR + MIN Group, *Scedosporium* infected larvae treated with VOR in combination with MIN; POS: Chlorhexidine; ITR: Itraconazole; VOR: Voriconazole. The experiment was repeated thrice on different days. ^*^P < 0.05; ^**^P < 0.01; ^****^P < 0.0001.

### Histopathological analyses

**Fig. 2:**
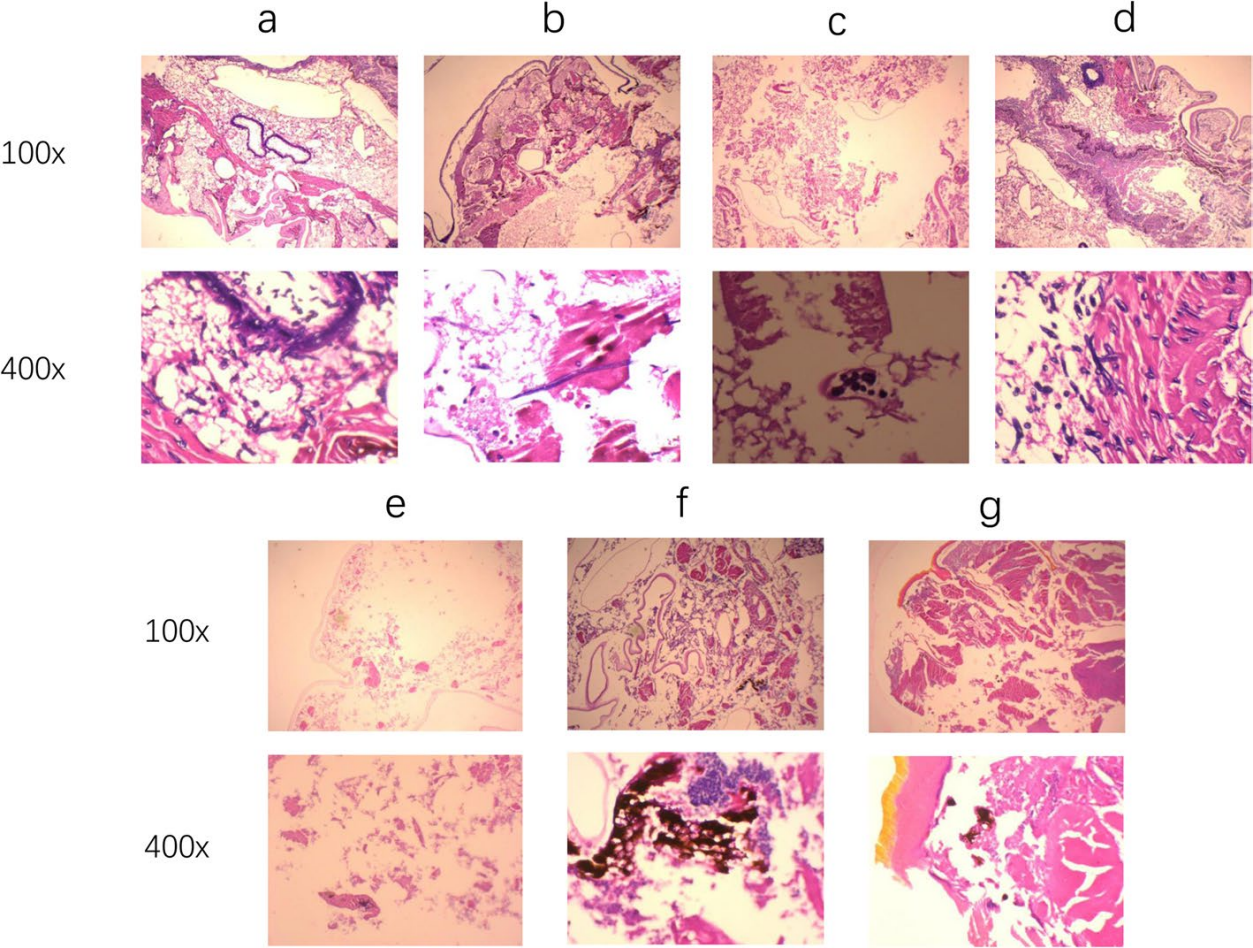
*G. mellonella* histopathology. **a** *Scedosporium* Group**; b** POS Group; **c** POS + MIN Group; **d** ITR Group; **e** ITR + MIN Group; **f** VOR Group; **g** VOR + MIN Group; Yellow and blue frame

We further performed a histopathological examination of the larvae. As depicted in Fig. 2, the formation of *Scedosporium apiospermum* spores and hyphae clusters in the infected tissues was detected in all the experimental groups. We observed that treatment with azoles alone resulted in a slight reduction in the number of visible fungal clusters relative to the *Scedosporium* group, while the number of visible fungal clusters was significantly decreased in the combination treatment groups as compared to the three azoles alone groups.

## Discussion

*Scedosporium* and *Lomentospora* species are important and very recalcitrant conditional pathogens. While the risk factors for infection of *Scedosporium* and *Lomentospora* species include a history of immunodeficiency, trauma, and drowning, the infection mainly involves skin, eyes, lungs, brain, even the whole body [4, 12]. Over the past decade, many trials have demonstrated that single azoles such as ITR, POS, VOR, and fluconazole exhibit certain activity against *Scedosporium* and *Lomentospora* species in vitro [13, 14]. The widespread use of azoles has led to a shift from azole-susceptible to azole-resistant infections. As a result, existing azoles are not ideal for the empirical treatment of infections with *Scedosporium* and *Lomentospora* species. Given the low antimicrobial activity of single drugs, combined use of multiple drugs may enhance their antimicrobial activities. Azole antifungal agents are commonly used in combination with other types of antifungal drugs in clinical practice, such as amphotericin B [15].

MIN has been used clinically as an antibiotic for many years since it was marketed. In recent years, MIN has gained an increasing attention with the research on its non-antimicrobial effect. Previous studies have shown that MIN reduces cell surface hydrophobicity and extracellular levels of 1,3-beta-D-glucan (1,3-BDG) in biofilms, revealing a mechanism underlying the inhibitory effect of MIN on *Candida albicans* [16]. Given that 1,3-BDG is the main component of fungal cell wall, it is suggested that MIN may possess an antifungal activity. Strikingly, MIN combined with low-dose fluconazole has been found to exert a synergistic antifungal effect [17, 18]. However, there have been few reports investigating the effect of MIN in combination with azoles against *Scedosporium* and *Lomentospora* species. In the present study, we examined the inhibitory effects of ITR, VOR, POS, and MIN alone as well as those of MIN in combination with azoles on *Scedosporium* and *Lomentospora* species.

Herein, a total of 16 isolates of *Scedosporium* and *Lomentospora* species were studied in vitro. While MIN alone showed no in vitro antifungal activity, MIN acted synergistically with ITR, VOR or POS against 8 (80%), 1 (10%), or 9 (90%) isolates tested, showing promising synergistic effects between MIN and azoles against *Scedosporium* and *Lomentospora* species. In addition, we found that *Lomentospora prolificans* displays low susceptibility to current antifungal drugs, being consistent with the previous reports [19, 20]. As listed in Table 1, we observed a significant decline in MICs of combination of MIN with ITR, VOR or POS against *Scedosporium* and *Lomentospora* species. The in vivo experiments provided more evidence that MIN acts synergistically with azoles against *Scedosporium* and *Lomentospora* species (Figs. 1 and 2). In agreement with a previous study showing that the presence of MIN increases susceptibility of pathogenic fungi to azoles, the present study did not identify any antagonism between MIN and azoles [21].

The combination of drugs is used mainly for increasing the efficacy of drugs or reducing their side effects. Fungal infections of the central nervous system (CNS) are characterized by the serious clinical manifestations as well as the difficulty in diagnosis and treatment, while *Scedosporium* infections affecting the CNS are relatively common [22, 23]. As a highly lipophilic molecule, MIN can easily enter cerebrospinal fluid and CNS through blood-brain barrier. Thus, it has potential for treating various CNS diseases [24, 25]. Overall, these observations may provide a theoretical basis for combined use of MIN and azoles in the treatment of fungal infections in CNS.

## Conclusions

In conclusion, MIN in combination with azoles may help to enhance the antifungal activities of azoles against *Scedosporium* and *Lomentospora* species, achieving a synergistic effect between MIN and azoles*.* Further studies need to be conducted for gaining insights into clinical impacts of the combination of MIN and azoles, as well as understanding clinical relevance of the in vitro data obtained in this study.

## Materials and methods

### Fungal strains

A total of 16 strains were included in the study. Among them, *Scedosporium aurantiacum* (CBS 116910), *Scedosporium minutispora* (CBS 116911), *Scedosporium boydii* (CBS 101.22), *Lomentospora prolificans* (CBS 467.74), *Scedosporium dehoogii* (CBS 117406), and *Scedosporium apiospermum* (CBS 116899) were generously provided by Prof. Sybren de Hoog of Fungal Biodiversity Centre (CBS) in Netherlands. The other strains were purchased from Nanjing Institute of Dermatology, China, including *Scedosporium boydii* (1), *Scedosporium boydii* (2), *Scedosporium boydii* (3), *Scedosporium apiospermum* (4), *Scedosporium apiospermum* (5), *Scedosporium apiospermum* (6), *Lomentospora prolificans* (7), *Scedosporium minutisporum* (8), *Scedosporium apiospermum* (9), and *Scedosporium boydii* (10). The species identity of each isolate was determined based on combined morphological/phenotypic characteristics and molecular sequencing of the internal transcribed spacer (ITS) ribosomal DNA (rDNA). Isolates were sub-cultured from frozen stocks on Sabouraud dextrose agar (SDA) prior to in vitro testing [26]. The authors declare that the ethical policies of the journal, as noted on the journal’s author guidelines page, have been adhered to, and the study was approved by the ethical review committee. The procedures were conducted in accordance with the US National Research Council’s guidelines for the Care and Use of Laboratory Animals.

### Antifungal agents

All tested agents, including ITR, VOR, POS, and MIN, were obtained from Selleck Chemicals (Houston, TX, USA) in powder form and dissolved according to the manufacturer’s protocol. The concentrations of tested agents were in the range from 0.5 to 64 mg/L for ITR, VOR and POS, or from 1 to 64 mg/L for MIN.

### In vitro susceptibility testing

In vitro interaction between MIN and azoles against *Scedosporium* and *Lomentospora* species was analysed using a microdilution checkerboard technique [27]. Conidia were harvested from cultures, grown for 3 days on SDA and then suspended in sterile distilled water containing 0.03% Triton. Thereafter, harvested conidia were adjusted to 1–5 × 10^6^ CFU/ml with a hemocytometer and then diluted 100 times with RPMI1640 broth containing 0.165 M MOPS (pH 7.0) according to the M38-A2 reference standard. Serial dilutions of tested agents were prepared by dilution with RPMI 1640. For preparation of test microplates, 50 μl of each concentration of azoles (ITR, VOR and POS) was added to columns 2 to 8, while 50 μl MIN was applied to rows 2 to 8. In each microplate, while row 1 and column 1 contained the azoles and MIN alone, respectively, column 9 was a drug-free well serving as the growth control. One hundred microlitre of fungal inoculum was applied to 96-well plates for a 72 h of incubation in ambient atmosphere at 35 °C, and the data were read visually. MIC values were defined as the lowest concentration of drugs at which the growth was completely inhibited. To assess the interaction outcomes, FICI value was calculated as follows: (MIC of Drug A in combination/MIC of Drug A alone) + (MIC of Drug B in combination/MIC of Drug B alone). The interaction of combined drugs was interpreted as FICI ≤0.5 for synergy, 0.5 < FICI< 4.0 for no interaction, or FICI≥4 for antagonism. All the tests were performed in triplicate.

### In vivo cytotoxicity assay

*G. mellonella* larvae were divided into nine groups (*N* = 20 per group): control group, Uninfected Control-Saline Treatment group, Infected Control-No Treatment group, MIN group, ITR group, VOR group, POS group, combined ITR and MIN group, combined VOR and MIN group, and combined POS and MIN group. We used several inoculation concentrations for infection. Since lower or higher concentrations resulted in low or high mortality in the control group, we used the optimal concentration for the observation. All nine groups except the control group and Uninfected Control-Saline Treatment group were injected with *S. apiospermum* (CBS 116899). *G. mellonella was pricked into the needle only, but not injected*. The experimental group was injected with 10ul of *S. apiospermum* (CBS 116899) solution at a concentration of 1 × 10^7^. *S. apiospermum* was injected into the last right proleg. Antifungal agents or a control solution (1 μg per larvae; drug concentration = 200 mg/L) was introduced after the area was cleaned with an alcohol swab. The control group, MIN group, ITR group, VOR group, POS group, combined ITR and MIN group, combined VOR and MIN group, and combined POS and MIN group were injected with 10ul of saline, 10ul MIN (12μg/ml), 10ul ITR (3μg/ml), 10ul VOR (0.75μg/ml), 10ul POS (1.5μg/ml), 10ul of mixed solution of MIN (12μg/ml) and ITR (3μg/ml), 10ul of a mixture of MIN (12μg/ml) and VOR (0.75μg/ml), and 10ul of mixed solution of MIN (12μg/ml) and POS (1.5μg/ml), respectively. The larvae survival was recorded daily for 6 days. The survival curve of larvae was analysed by Kaplan-Meier method, and the difference was determined by Mantel Cox test. *P* < 0.05 was considered statistically significant.

Larval tissues were collected, fixed in 10% neutral formalin and then dehydrated using an ethanol gradient. Thereafter, tissue samples were embedded in paraffin and xylene, sectioned at a thickness of 8 μm and stained with hematoxylin and eosin (HE). Finally, stained sections were evaluated with FSX100 fluorescence microscopy at magnifications of 10× and 40 × .

## Data Availability

The data that support the findings of this study are available from the corresponding author upon reasonable request.

## Abbreviations

ITR: Itraconazole
VOR: Voriconazole
POS: Posaconazole
MIN: Minocycline
CNS: Central nervous system
MIC: Minimum inhibitory concentration
1,3-BDG: 1,3-beta-D-glucan
FICI: Fractional inhibitory concentration index
HE: Hematoxylin and eosin
